# Role of *Secretoglobin*^+^ (club cell) NFκB/RelA-TGFβ signaling in aero-allergen-induced epithelial plasticity and subepithelial myofibroblast transdifferentiation

**DOI:** 10.1186/s12931-021-01910-w

**Published:** 2021-12-20

**Authors:** Melissa E. Skibba, Xiaofang Xu, Kurt Weiss, Jan Huisken, Allan R. Brasier

**Affiliations:** 1 School of Medicine and Public Health, University of Wisconsin Madison, 4248 Health Sciences Learning Center, Madison, WI 53705 USA; 2 Morgridge Institute for Research, Madison, WI USA; 3 Dept. of Integrative Biology, University of Wisconsin, Madison, WI USA; 4 Institute for Clinical and Translational Research, Madison, WI USA

**Keywords:** TGFβ, EMT, RELA, Epithelial plasticity, Aeroallergen, Cat dander

## Abstract

Repetitive aeroallergen exposure is linked to sensitization and airway remodeling through incompletely understood mechanisms. In this study, we examine the dynamic mucosal response to cat dander extract (CDE), a ubiquitous aero-allergen linked to remodeling, sensitization and asthma. We find that daily exposure of CDE in naïve C57BL/6 mice activates innate neutrophilic inflammation followed by transition to a lymphocytic response associated with waves of mucosal transforming growth factor (TGF) isoform expression. In parallel, enhanced bronchiolar Smad3 expression and accumulation of phospho-SMAD3 was observed, indicating paracrine activation of canonical TGFβR signaling. CDE exposure similarly triggered epithelial cell plasticity, associated with expression of mesenchymal regulatory factors (*Snai1* and *Zeb1*), reduction of epithelial markers (*Cdh1*) and activation of the NFκB/RelA transcriptional activator. To determine whether NFκB functionally mediates CDE-induced growth factor response, mice were stimulated with CDE in the absence or presence of a selective IKK inhibitor. IKK inhibition substantially reduced the level of CDE-induced TGFβ1 expression, pSMAD3 accumulation, *Snai1* and *Zeb1* expression. Activation of epithelial plasticity was demonstrated by flow cytometry in whole lung homogenates, where CDE induces accumulation of SMA^+^Epcam^+^ population. Club cells are important sources of cytokine and growth factor production. To determine whether Club cell innate signaling through NFκB/RelA mediated CDE induced TGFβ signaling, we depleted RelA in *Secretoglobin* (*Scgb1a1*)-expressing bronchiolar cells. Immunofluorescence-optical clearing light sheet microscopy showed a punctate distribution of Scgb1a1 progenitors throughout the small airway. We found that RelA depletion in Secretoglobin^+^ cells results in inhibition of the mucosal TGFβ response, blockade of EMT and reduced subepithelial myofibroblast expansion. We conclude that the Secretoglobin—derived bronchiolar cell is central to coordinating the innate response required for mucosal TGFβ1 response, EMT and myofibroblast expansion. These data have important mechanistic implications for how aero-allergens trigger mucosal injury response and remodeling in the small airway.

**Supplementary Information:**

The online version contains supplementary material available at 10.1186/s12931-021-01910-w.

## Background

Allergic asthma (AA) is a global health concern for 235 million people worldwide [1] who are affected by consequences of airway remodeling, Th2 polarization and eosinophilia [2]. Remodeling is the earliest abnormalities in AA [3, 4], a collective term referring to airway epithelial repair [epithelial mesenchymal transition (EMT)] and cell state transitions including myofibroblast activity, metaplasia and smooth muscle hypertrophy [1, 5]. Repetitive allergic exacerbations produce reduction of lung function [6–8]. The detailed mechanisms how aeroallergens coordinate this complex tissue response are largely unknown.

Although the final consequence of allergic sensitization is the activation of Th2 immunity and production of allergen-specific IgE, a close interaction with structural cells and activation of the innate immune response is important. A body of work has shown that aero-allergens are plant and animal-derived products that modify the epithelial barrier function and activate innate signaling cascades [9]. For example, the extensively studied *Dermatophagtoides pteronyssius* [house dust mite (HDM)] is a common complex aeroallergen containing bacterial cell wall products [lipopolysaccharide (LPS) and β-glucan [10] as well as mite-produced proteases [11]. The *Dermatophagtoides*-produced cysteine protease allergen, Der p1, disrupts epithelial tight junctions by cleavage of zona occludens, and activates release of pro-inflammatory cytokines by epithelial cells and plasticity [12]. At the mechanistic level, Der p1 activates the protease-activated receptor (PAR)-2 cleaving its NH_2_ terminus, irreversibly activating signaling [13]. The cockroach allergen, Per a 10, also induces innate signaling by protease activity directed to PAR-2 [14]. In contrast, Ragweed pollen contains an endogenous NADPH-driven oxidase that disrupts the epithelial barrier by formation of reactive oxygen species [15], producing CXCL2 release and neutrophilic inflammation [16]. The alkaline protease 1 (Alp1) is an *Aspergillus* spp.-derived aeroallergen that disrupts epithelial tight junctions by cleaving epithelial cadherin, disrupting barrier function, producing IL33/CCL2 secretion and eosinophilia [17]. Collectively, these distinct aero-allergens induce mucosal signaling by self-contained enzymatic activity triggering epithelial expression of DC and neutrophil activating cytokines.

The focus of this study, cat dander, represents one of the most common aero-allergens associated with AA [18], with > 25% patients demonstrating sensitization to the major cat allergen, Fel d1 [19]. Contrary to the aeroallergens described earlier, cat dander is a mixture of cat allergens (Fel d1-Fel d8), but lacks significant LPS [20] and/or protease activity [21]. Our earlier work has shown that cat dander functions as a potent innate stimulus, activating the MD2 co-receptor and TLR4, upstream of the Myd88-NFκB pathway [16, 20] producing CXCL2 secretion and neutrophil recruitment [20]. Importantly, CDE-induced innate inflammation activates TLR4-MD2 through a *CD14-independent* pathway, providing additional evidence that CDE-induced inflammation is LPS-independent [16, 20, 21]. Consequently, further examination of the mechanisms of signaling and allergy will reveal an important new understanding of the complex interactions between aeroallergens, sensitization, and AA.

Th2 sensitization is associated with remodeling of the structural components of the airway, including expansion of the *lamina reticularis*, epithelial cell-state changes, and infiltration of tissue-resident eosinophils [5, 22]. Induction of epithelial plasticity, a term inclusive of epithelial cell-state changes known as mesenchymal transition (EMT) and partial EMT are an important component of remodeling in AA [23]. In this dynamic dedifferentiation program, expression of epithelial cadherin (CDH1) is repressed, producing epithelial barrier disruption, and expression of extracellular matrix (ECM)-remodeling proteins are activated, producing changes in the *lamina reticularis* [24]. In Th2 models, epithelial IKK signaling is linked to cytokine expression and fibrosis in response to OVA sensitization [25] as well as mucosal aeroallergen exposure [6]. In addition, transforming growth factor β (TGFβ)-1 produced by tissue-resident eosinophils initiates epithelial plasticity in the atopic epithelium [24]. However, the relationship between NFκB signaling, epithelial plasticity and TGFβ production is not fully understood. The findings that the majority of TGFβ1-producing cells in bronchial specimens from severe asthma are eosinophils [26] indicate that, in established atopy, maintenance of EMT/pEMT is mediated by infiltrating eosinophils.

The question on how aero-allergen exposure produces epithelial plasticity *prior* to eosinophil infiltration and atopy is incompletely explored. Previous work by others have shown that HDM exposure triggers epithelial TGFβ expression and plasticity through Der p1 protease activity [12] and that *inducible* expression of TGFβ in airway epithelial cells plays a central role in HDM sensitization [27]. However, because of the distinct pathways involved in HDM protease activation, these studies do not inform understanding of CDE-induced remodeling.

Here, we tested the hypothesis that CDE activates mucosal growth factor responses driving airway remodeling. Specifically, our focus on the sentinel role of the epithelial *Secretoglobin* (Scgb1a1)-expressing “Club” cell. Club cells are a major sources of innate neutrophilic cytokine responses to virus infections and viral patterns [28, 29]. Additionally, club cells produce the dendritic cell-activating cytokines IL-25 and -33 in response to the *Aspergillosis* Alp1 protease [17]. Our study indicates that CDE activates epithelial TGFβ synthesis through the RelA pathway in *Secretoglobin-*expressing Club cells, triggering epithelial plasticity and expansion of subepithelial myofibroblasts. These findings suggest that Scgb1a1-producing epithelial cells act as “central sensors” for aero-allergen exposure, through TGFβ signaling and airway remodeling.

## Materials and methods

### Cat dander extract (CDE)

Lyophilized CDE was purchased from Stallergens/Greer Labs (Lenoir, NC). We reported earlier that these extracts had less than 0.1 pg endotoxin/1 μg protein using LAL chromogenic endotoxin quantitation kit (Thermo Scientific, Hudson, NH)[16].

### hSAEC culture and RelA-silencing

Human small airway epithelial cells (hSAECs) were stimulated in the absence or presence of CDE (20 µg, 5 days). For RNA silencing, hSAECs expressing Dox-regulated RelA shRNA were established using puromycin selection [30]. hSAECs were incubated in the absence or presence of doxycycline (Dox, 2 μg/ml, 5 days) and stimulated with CDE (20 µg, 5 days). Samples were collected for mRNA and chromatin immunoprecipitation.

### Animal study

Animal experiments were performed according to the NIH Guide for Care and Use of Experimental Animals and approved by the University of Wisconsin-Madison Institutional Animal Care and Use Committee (approval no. 1312058A). 6-week old C57BL/J6 black mice were administered CDE (80 µg/kg diluted in PBS) or PBS (control mice) every day for 1–4 days and euthanized at each day (i.e. 1, 2, 3, 4). For the IKK inhibitor, the animals were treated every day with CDE or PBS and treated with IKK inhibitor (BMS345541; 10 mg/kg via IP) every day for 5 consecutive days before being euthanized. For all studies, bronchoalveolar lavage fluids (BALF) and lung tissues were collected for ELISA, immunofluorescence staining, and qRT-PCR analysis.

RelA depletion in the *Scgb1a1*^CreER^™^/+^ × RelA^fl/fl^ mouse [28] was achieved by tamoxifen (TMX) injection. Both male and female mice aged 3 to 4 weeks old were used. After 3 weeks, mice were exposed to CDE (80 µg/kg) for 4 consecutive days via intranasal administration. Mice were euthanized and lung tissue was collected for bronchoalveolar lavage fluid (BALF), histology, and qRT-PCR analysis. For imaging of whole lung, *Scgb1a1*^CreER^™^/+^ × RelA^fl/fl^ mice [28] were crossed with mTmG (*Gt(ROSA)26Sor*^*tm4(ACTB−tdTomato,−EGFP)Luo*^), The Jackson Laboratory, Bar Harbor, ME). Whole lungs from *Scgb1a1*^CreER^™^/+^ × RelA^fl/fl^ X mTmG were removed, cleared and immunostained.

### Tamoxifen (TMX) administration

TMX was from Sigma (T5648). For administration, TMX was dissolved in 10% ethanol and 90% corn oil for a 10-mg/ml working solution. TMX was administered at a dose of 1 mg/day via the intraperitoneal route for 10 days.

### Cell Count and differentials

Bronchoalveolar lavage fluids (BALF) was spun down at 1000xg using ALLEGRA X-30R centrifuge (Beckman Coulter Life Sciences, Indianapolis, IN). Supernatant was discarded and red blood cell lysis buffer (Qiagen, Germantown, MD) was added and the cells were spun down again at 1000xg with the supernatant discarded. PBS with 3% bovine serum was added and the cells were counted using a TC20 automated cell counter (BioRad, Hercules, CA). Total cell count was calculated against to the amount of BALF per sample.

Differentials were obtained by spinning down the cells onto Superfrost™ Plus microscope slides (ThermoFisher Scientific, Waltham, MA) using the StatSpin® CytoFuge® 2 (HemoCue, Ängelholm, Sweden) according to manufacturer’s recommendations. Slides were fixed with methanol and then stained with Wright Giemsa (ThermoFisher Scientific) according to the manufacturer’s instructions. Immediately after the slides were stained, they were photographed and counted using the Echo Revolve (ECHO, San Diego, CA) at × 60. The calculation of macrophages, lymphocytes, neutrophils and eosinophils are a representation of 200 cells counted compared the total cell count of the complementary sample.

### TGFβ1 ELISA assay

BALF was obtained from mouse lung by flushing the lungs with PBS and spinning down the fluid to remove the cells. A Mouse TGF-beta 1 DuoSet ELISA (R&D Systems, Minneapolis, MN) was used according to manufacturer’s instruction to measure immunoreactive TGβ1.

### Immunostaining

Whole lungs were fixed in 10% (vol/vol) neutral buffered formalin for 1 day, processed into paraffin blocks, and cut into 4-μm sections for hematoxylin and eosin (H&E). Immunofluorescence sections were rehydrated with xylene and serial concentrations of ethanol. Antigen retrieval was obtained using a citrate buffer (Thermofisher, Waltham, MA). Sections were blocked with a diluted normal goat serum before incubation with primary antibodies: Anti-TGFβ1 (Abcam, 1:200), NfĸB-p65 (RelA, Bioss, 1:200), Green fluorescent protein (GFP, Abcam, 1:100), Snail (Cell Signaling, 1:200), alpha-SMA (Abcam, 1:200), and COL1A1 (Cell Signaling, 1:200); and secondary anti-rabbit or anti-mouse Alexa Fluor 647 (ThermoFisher Scientific, 1:1000) and/or anti-mouse Alexa Fluor 561 (ThermoFisher Scientific, 1:1000). All paraffinized fluorescent stained slides were mounted using ProLong™ Gold Antifade Mountant with DAPI (ThermoFisher Scientific). Imaging was performed using a Nikon A1RS Confocal Microscope with a × 20 magnification. Quantification and analysis were done by measuring the nuclear intensity of positive cells surrounding the major airways with ImageJ software. Alternatively for COLA1 and alpha-SMA, the alpha-SMA^+^ were superimposed onto the COL1A1^+^ to measure the myofibroblast-positive intensity using ImageJ software. Data was expressed as a mean of scores recorded by two blinded investigators.

### Q-RT-PCR

For animal experiments, the right lung was homogenized using the Mini Bead Mill Homogenizer (VWR, Radnor, PA) with 1.4 mm ceramic beads (Omni International, Kennesaw, GA). Total RNA was extracted using the RNeasy Mini Kit (Qiagen, Germantown, MD) according to manufacturer’s instructions. RNA was reverse transcribed using SuperScript IV (ThermoFisher Scientific) according to manufacturer’s instructions. cDNA was amplified using iTaq™ Universal SYBR® Green Supermix (Bio-Rad, Hercules, California) and gene-specific mouse primers. Alternatively, human Taqman™ (ThermoFisher Scientific) and gene-specific human primers used TaqMan™ Universal PCR Master Mix (ThermoFisher Scientific) to amplify the cDNA. All samples were run using the AriaMx Realtime PCR System (Agilent Technologies, Santa Clara, CA). Relative changes in gene expression were quantified relative to control GADPH or PPIA transcripts, respectively, using the ΔΔC_t_ method [31]. The forward and reverse gene-specific Q-RT-PCR primers are listed in Additional file 1: Table S1.

### Immunofluorescent-optical clearing light sheet microscopy

Whole lungs were extracted from the mouse and fixed with 4% Formaldehyde. To stain the lungs, the tissue was first perfused with blocking buffer (5% goat serum), similar to our normal IHC, overnight using an intact catheter into the trachea and then incubated with anti-GFP Ab [1:100 in PBS and 1% Triton X (Sigma)] for 4 days. Tissue was perfused in PBS and 1% Triton X before secondary anti-rabbit Alexa Fluor 647 Ab was perfused into the lung for 2 days. Lastly, the lung was washed before being cleared by progressive dehydration (methanol) and then a 1:2 vol/vol mixture of benzyl alcohol/benzyl benzoate, rendering them optically transparent where in the catheter was removed and lungs were imaged.

Image data was acquired on a custom-built light sheet microscope designed for imaging large optically cleared samples. A multi-channel coherent laser source (Omicron sole-6) is collimated and expanded to achieve the required light sheet size for the 4 mm × 4 mm field of view and 20 μm sheet waist for optical sectioning. The light sheet is formed with a cylindrical lens and resonant mirrors (EOPC) pivot the individual light sheets for stripe suppression [32]. The system uses long working distance air lenses for dual-sided illumination (Nikon Plan Fluor 4x/0.13). Both illumination and detection lenses are moveable to adjust for sample size, clearing media refractive index (RI), and chromatic aberration.

Each lung was mounted by clamping the main airways suspended in a custom 40 mm × 40 mm quartz sample chamber (FireFlySci, Brooklyn, NY) and translated through the light sheet with a set of precision motors (Physik Instrumente, M-111.1DG). The detection lens (AZ-Plan Apo 2 × NA/0.2) is paired with a 150 mm tube lens for × 3 magnification and images were acquired with a 16MP sCMOS camera (Photometrics Iris 15). The 3D image volumes were generated at multiple positions to cover the sample and tiles were stitched using the Fiji plugin BigStitcher [33]. The final image volumes were approximately 1 TB per lung.

### Lung single cell suspension and flow cytometry

Lung of the right lobe was harvested from 7-week old C57BL/6 black mice treated with vehicle (PBS) or CDE (80 µg/kg) for 4 consecutive days. Before removal, the lungs were filled with about 1 ml digestion buffer, comprised of 100 mg/ml Dispase II (Roche Diagnostics, Indianapolis, IN), 10 mg/ml Collagenase A (Roche Diagnostics, Indianapolis, IN), 1500 kU/ml DNase I (Sigma-Aldrich, St. Louis, MO), and 0.025 M CaCl_2_ before being mechanically minced and incubated at 37C for 30 min. The suspension was filtered through a 40 µm filter into a 50 ml Falcon tube. The suspension was centrifuged at 900 g for 5 min and the supernatant was aspirated. The pellet was resuspended in 405 Live/Dead dye (Invitrogen, Waltham, MA), according to the manufactures instructions, and incubated in the dark at room temperature for 30 min. PBS was added to the suspension, centrifuged at 900 g for 5 min, and the supernatant was aspirated. Cells were fixed in 4% paraformaldehyde for 10 min, followed by an additional washing step used for staining. Fc-receptors were first blocked by anti-CD16/CD32 antibodies (Biolegend, San Diego, CA) for 10 min a solution of 0.1% saponin (Sigma-Aldrich, St. Louis) and 1% BSA (Sigma-Aldrich, St. Louis). After the pre-incubation, a cocktail of the following antibodies were added: anti-CD45-Alexa Fluor BV605 (Biolegend, San Diego, CA, Cat# 103139, 1/1000 dilution), anti-aSMA-FITC (Sigma-Aldrich, St. Louis, MO, Clone: 1A4, Cat# F3777, 1/1000 dilution), and anti-mouse CD326-PE (Biolegend, San Diego, CA, Cat# 118205, 1/1000 dilution). Antibody cocktails were prepared in 0.1% saponin, 1% BSA buffer containing Fc-block. Following a 30 min incubation, cells were washed with 0.1% saponin and 1% BSA buffer, centrifuged at 500 g for 5 min, and aspirated and resuspended in FACSFlow buffer to be run for flow. Gating was performed using Fluorescence Minus One (FMO) controls for each antibody (Additional file 1: Fig. S3) and samples were quantified using the percent of parent. All samples were run on the Attune NxT (Thermofisher) and analyzed with FlowJo V10.8.0.

### Two-step chromatin immunoprecipitation (XChIP) assays

XChIP was performed as described previously [34]. In brief, hSAECs were washed twice with PBS. Cross-linked chromatin was fragmented by sonication, and equal amounts were immunoprecipitated with protein A magnetic beads (Dynal Inc.). Eluted DNA was de-crosslinked, purified and quantitated using gene-specific primers amplifying the *TGFβ1* promoter. Data is presented as the technical replicates using a fold-change relative to the IgG after normalization to input chromatin [34].

### Statistical analysis

Differences among the means were tested for significance by ANOVA with Fisher’s least significance difference test. Significance was reached when the p-value was 0.05. Values presented are means ± standard deviation (S.D.).

## Results

### CDE induces waves of inflammation and mucosal TGFβ isoform expression

**Fig. 1:**
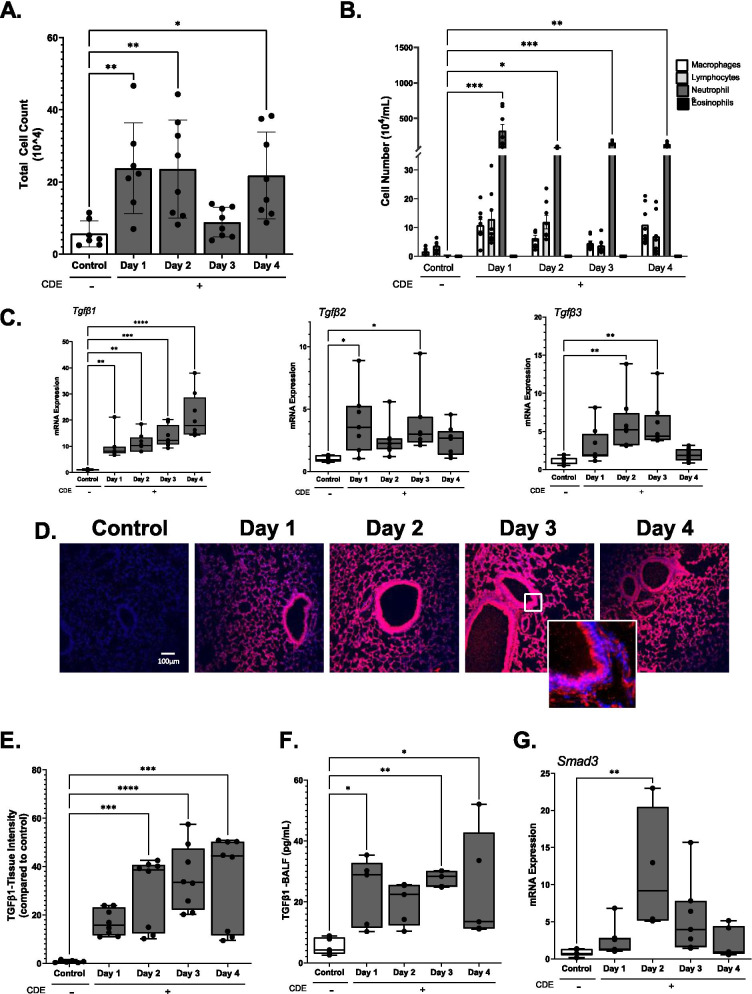
Cat dander extract induces mucosal TGFβ. C57BL/6J mice were treated with daily challenges of CDE or PBS i.n. over 4 days. **A** Cell count of bronchoalveolar fluid (BALF) from mouse lung. Mean ± S.D. of BALF total cell counts (10^4^/ml) at each time point for n = 6–8 animals/group (each symbol represents an independent animal). *p < 0.05; **p < 0.01; post hoc t-test for indicated contrast. **B** Differentials of BALF cell counts. Mean ± S.D. (10^4^/ml) for macrophages, lymphocytes or neutrophils at each time point. *p < 0.05; **p < 0.01; ***p < 0.001 post hoc t-test for indicated contrast. No eosinophils were detected. **C** Time dependent expression of *Tgfβ-1/2/3* mRNAs. Mean ± S.D.of fold change abundance for each mRNA mRNA by Q-RT-PCR of total mouse lung RNA. n = 6–8 animals/group. **D** Immunofluorescence microscopy (IFM). Sections were stained with anti-TGFβ1 antibody (Ab, red color) and counter-stained with DAPI (blue) to visualize nuclei. Representative images shown at 20X magnification with selected enhanced image of the epithelium. **E** Quantitation of TGFβ1. Fluorescence images were quantified by FIJI. Mean ± SD of fluorescence intensity (FI) for n = 6–8 animals/group. **F** Bioactive TGFβ1 abundance in BALF. Shown are mean ± S.D. of free TGFβ1 levels in ELISA (n = 5 animals/group). **G** Induction of *Smad3*. Relative fold changes in *Smad3* mRNA by Q-RT-PCR; n = 6–8 animals/group

Although the induction of airway inflammation-remodeling in response to protease-encoded aero-allergens is known, little is known about early steps in the initiation of CDE-induced remodeling. To address this knowledge gap, we conducted a time-course study of daily Cat Dander Extract (CDE) in naïve C57BL/6 mice. After 1 days of intranasal CDE exposure, a rapid induction of neutrophils in the BALF was observed, that persisted for 3 days, then decreased, replaced by a second wave of macrophages and lymphocytes (Fig. 1A, B). No eosinophils were induced, indicating that these changes are reflective of innate responses.

Previous work using HDM has shown that *inducible* epithelial TGFβ1 expression plays an important role in expansion of a population of luminal innate lymphoid cells (ICL)2s, linked to airway hyperreactivity and sensitization [27]. However, the inducible epithelial TGFβ response is highly stimulus-dependent [27], and whether CDE induces growth factor responses in unsensitized mucosa is unknown. To determine whether CDE activates TGFβ isoform expression, total lung RNA was isolated from animals in the time course, and changes in *Tgfβ -1, -2* and *-3* mRNA abundance were assessed by Q-RT-PCR. The expression of *Tgfβ1* was induced tenfold (14 ± 5, p < 0.01, post-hoc T-test, n = 6–8 animals) 1 days after CDE challenge that remained stable until the 4^th^ challenge, when a second inflection was observed, concomitantly with the influx of macrophages and lymphocytes (Fig. 1C). *Tgfβ2* and *Tgfβ3* mRNAs were also rapidly induced, but in contrast to *Tgfβ1*, were induced to a much smaller degree and their induction was transient, declining after 4 days (Fig. 1C).

### CDE produces bioactive TGFβ release into the BALF

Recognizing that TGFβ is sequestered in extracellular matrix stores, we asked whether bioactive TGFβ1 was produced by CDE exposure. For this purpose, we stained for free TGFβ1 in the tissue slides. Here a robust accumulation of TGFβ1 protein was observed, peaking on d 3 with a 38-fold increase in signal intensity (Fig. 1D, E). We further confirmed the presence of activated TGFβ1 protein in the BALF by ELISA. After 1 d of CDE stimulation, free TGFβ1 increased from 5.4 ± 2.5 to 24 ± 10.1 pg/ml (p < 0.05 post hoc, n = 5 animals/group, Fig. 1F). These data indicate that inducible TGFβ1 synthesis was coupled with release of free TGFβ1 in the airway tissue.

### CDE activates canonical mucosal TGFBR signaling

We next sought to determine whether the free TGFβ was actively signaling. TGFβ1 signals by binding to TGFβRII, a transmembrane serine-threonine kinase, whose activation recruits cytoplasmic SMAD3, a transcription factor that is positive autoregulated, mediating the canonical TGFβ response [24]. We observed an increased expression of *Smad3* mRNA in the airway with a peak of 14 ± 11-fold at 2 days, p < 0.01, post-hoc t-test, n = 6–8 animals) (Fig. 1G), and concluded that CDE induces local paracrine activation of TGFβ signaling.

### CDE induces epithelial plasticity

**Fig. 2:**
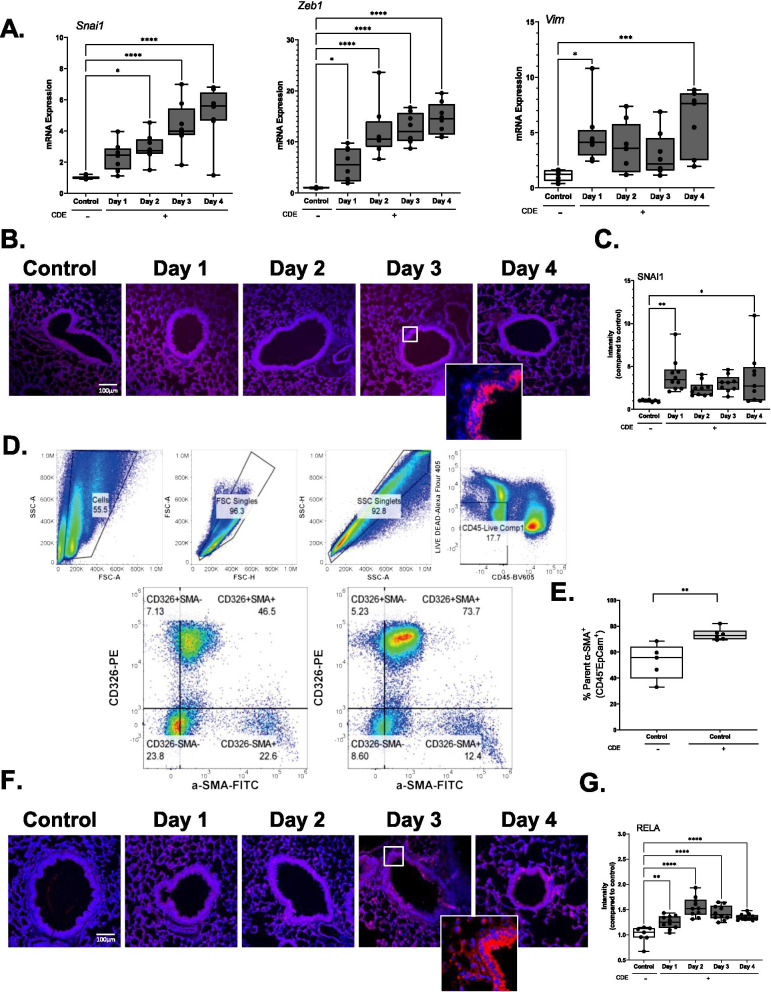
Cat dander extract induces epithelial plasticity and activation of NFκB/RELA. C57BL/6J mice were treated with daily challenges of CDE or PBS i.n. over 4 days. **A** Changes in mesenchymal markers. Relative fold changes of indicated transcripts by Q-RT-PCR of total lung RNA; *p < 0.05, compared to Control, n = 6–8/group, t-test. **B** IFM. Sections were stained with anti-SNAI1 Ab (red) and DAPI (blue) for nuclear staining. Representative images shown at × 20 with selected enhanced image of the epithelium. **C** Relative fold changes in SNAI1 FI for each treatment group (n = 6–8 animals/group). **D** Single cell flow cytometry using a 4-day CDE exposure model. Representative gating of cell selection, removal of debris, doublets and Live-CD45^−^ cells. [Left] a representation of SMA^+^ and CD326^+^ control cells vs [Right] SMA^+^ and CD326^+^ CDE-exposed cells. **E** % SMA^+^ and CD326^+^ cells in the 4-day CDE exposure model, n = 5. **F** Sections stained with anti-RELA Ab (red) and DAPI (blue) for nuclear staining. Representative images shown at × 20 with selected enhanced image of the epithelium. **G** Relative fold changes in RELA FI for each treatment group (n = 6–8 animals/group)

In non-transformed epithelial cells, TGFβ activates epithelial plasticity [35], a cell-state transition associated with loss of apical polarization, loss of adhesion complexes and expression of mesenchymal genes. TGFβ regulates mesenchymal gene transcription through SMAD-dependent and SMAD-independent TGFβ receptor signaling pathways [24, 36]. In the SMAD-dependent pathway, activated SMAD2/3 forms complexes with SMAD4 in the nucleus, inducing its autoregulation and activating expression of core EMT-promoting transcription factors such as the Snail Family Transcriptional Repressor (SNAI)-1 and -2) and Zinc Finger E-Box Binding Homeobox (ZEB)-1 and 2 and cascades of downstream mesenchymal genes, including the intermediate cytoskeletal protein vimentin (VIM) [24, 37]. Expression of the EMT regulators were first quantitated by Q-RT-PCR. We observed that *Snai1* mRNA dramatically increased 3 ± 1 -fold (p < 0.01, post-hoc T-test, n = 6–8 animals/group) after 1 day of CDE exposure and continued to accumulate, rising to a 5 ± 3.0-fold after the 4th exposure (Fig. 2A). Similarly, *Zeb1* mRNA was increased 5 ± 3.0-fold after the first day and continued to increase to 14 ± 3.0-fold after the 4th days of exposure (p < 0.01, post-hoc T-test, n = 6–8 animals, Fig. 2A). Likewise, *Vim* mRNA showed a biphasic induction with an increase of 4 ± 3.0-fold after the first exposure, falling to 2.0-fold on day 3 and then increasing again on the 4th exposure (Fig. 2A).

Immunofluorescence microscopy was performed for SNAI1, which revealed threefold increase in SNAI1 abundance from d1–d4 in the small airway mucosa and alveoli (Fig. 2B, C).

Because the resolution of microscopy could not definitively demonstrate that the epithelial cells were acquiring mesenchymal characteristics, we conducted flow cytometry assays with epithelial selective markers. Mice were challenged with CDE for 4 days and single cell isolates from whole lung homogenates were stained with CD45, CD326/Epcam and alpha smooth muscle cell actin (αSMA). αSMA was in quantitated lacking CD45 expression (leukocytes) and positive for expression of Epcam (epithelial cells) (Fig. 2D). We observed a significant twofold increase in the αSMA^+^/CD45^−^/Epcam^+^ population (p < 0.01, n = 5 animals, Fig. 2E). These data support that CDE triggers epithelial plasticity.

### CDE activates NFκB signaling

Because chronic CDE-induced inflammation activates NFκB signaling in sensitized animals [6], we next sought to determine whether NFκB was activated rapidly by CDE in naïve animals. Mucosa of C57BL/6 mice exposed to the CDE time course were stained with anti-NFκB/RELA antibodies. We observed CDE enhanced the expression of RELA in a similar distribution as that of SNAI1, peaking at 2 days (1.6 ± 0.2, p < 0.01, post-hoc t-test, n = 6–8 animals, Fig. 2F, G). Enhanced RELA expression is a characteristic of NFκB activation in response to diverse conditions [28, 30, 38].

### IKK signaling is required for canonical TGFβ signaling and epithelial plasticity

In airway epithelial cells, RELA exists in the cytoplasm as an inactive complex bound to IκB [38]. NFκB activation is mediated by the IκB kinase (IKK) complex whose phosphorylation of IκB triggers its proteasomal degradation, releasing sequestered RELA to enter the nucleus [39, 40]. To initially determine whether IKK-NFκB signaling mediates the TGFβ mucosal response, C57BL/6 mice were randomized to receive a 4-day CDE challenge in the absence or presence of the allosteric IKK inhibitor (IKKi), BMS345541 [41]. BMS345541 is highly selective for catalytic subunits of IKKi with an IC50 of 4 μM without activity on 15 closely related mitogen-activated protein kinases [41].

**Fig. 3:**
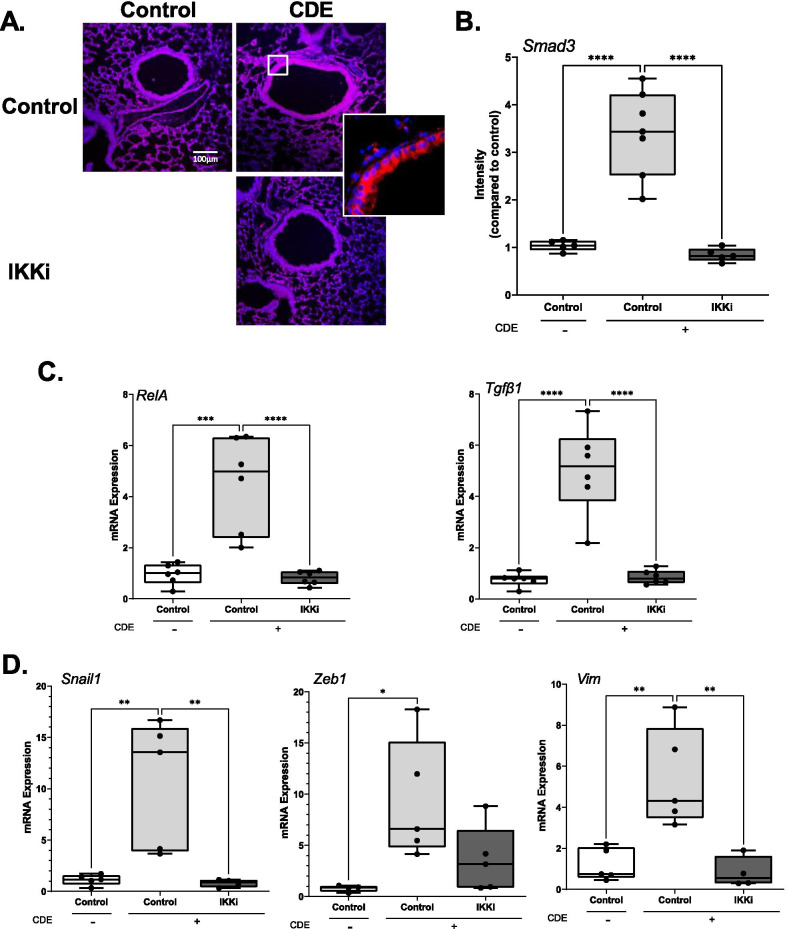
Mucosal TGFβ signaling is regulated by IKK. C57BL/6 J mice were treated with daily challenges of CDE or PBS i.n. and given BMS-345541 (IKKi) i.p. simultaneously over 5 days, n = 5–6. **A** IFM. Sections were stained with anti p-SMAD3 Ab (red) and DAPI (blue). Representative images shown at × 20 magnification, with selected enhanced image of the epithelium. **B** Quantitation of p-SMAD3. Relative fold changes in FI for each treatment group. **C** mRNA abundance. Relative fold changes in *RelA* and *Tgfβ1* mRNA abundance in total lung by Q-RT-PCR. **D** Changes in mesenchymal gene expression. Relative fold changes in *Snai1*, *Zeb1*, and *Vim* mRNAs in total lung RNA; *p < 0.05 compared to Control, t-test

In the CDE-challenged mice treated with IKKi, we noted that the 3.4-fold induction of pSMAD3 was substantially reduced to less than control levels (Fig. 3A, B). In parallel, the CDE-enhanced expression of *RelA* and *Tgfβ1* were all reduced compared to control levels (Fig. 3C). To determine whether IKK signaling also induced epithelial plasticity, we measured expression of mesenchymal genes. We observed that CDE activated *Snai1* 13-fold, *Zeb1* sixfold, and *Vim* 4.2-fold (Fig. 3). All these inductions were markedly reduced with the IKK inhibitor (Fig. 3D). These data indicated that the canonical NFκB pathway mediates CDE-induced TGFβ1 expression, paracrine signaling and downstream plasticity.

### CDE induces epithelial plasticity in human Club cell progenitors

**Fig. 4:**
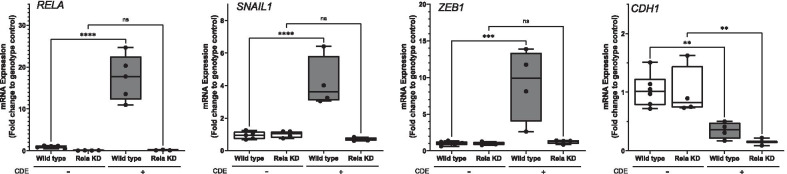
RelA-knock down (KD) reduces mucosal TGFβ by attenuating EMT. RelA shRNA-expressing hSAECs under control of a Dox inducible promoter were cultured in the presence of DOX for 7 days and then treated in the absence or presence of CDE for 4 d. Abundance of *RelA, SNAI1, ZEB1* and *CDH1* mRNAs were determined by Q-RT-PCR. *p < 0.05, post hoc t-test for n = 4–6 biological replicates. The basal levels of *SNAI1* and *ZEB1* were affected by RELA depletion, an expected finding because RELA directly binds to *SNAI1* and *ZEB1* promoters [8], and functions as an epigenetic suppressor in the absence of pathway activation [65] To control for this, data are normalized to each genotype untreated control

To confirm that CDE induced epithelial plasticity, we conducted stimulation experiments in human small airway epithelial cells (hSAECs) that represent Club-cell progenitors [hSAECs express *SCGB1A1* [42]. hSAECs expressing a Dox-inducible RelA shRNA [30] were treated for 7 days ± Dox to induce RELA depletion and then stimulated ± CDE for 4 additional days. The cells were then prepared for Q-RT-PCR analysis of mesenchymal and differentiated epithelial gene expression. In the Wild type cells, observed that CDE induced a 16.5 ± 1.9-fold increase in RELA mRNA (p < 0.01, n = 4–6 biological replicates, t-test, Fig. 4), a 4.2 ± 0.55-fold increase in SNAI1 expression (p < 0.01, n = 4–6 biological replicates, t-test, Fig. 4), and a 9.1 ± 1.7-fold increase in ZEB1 mRNA expression (p < 0.01, t-test, n = 4–6 replicates, Fig. 4). In parallel, CDE produced a substantial reduction in epithelial cadherin (CDH1) expression in the wild type cells to 0.35 ± 0.2-fold decrease in CDH1 expression relative to untreated cells (p < 0.01, t-test, n = 4–6 replicates, Fig. 4). Collectively, the increase in mesenchymal core transcription factor expression and reduction of the epithelial differentiation marker are sine qua non of epithelial plasticity.

### NFκB/RELA mediates CDE induced activation of the core mesenchymal transcription factors

The Doxtreated cells were then examined to determine whether NFκB/RelA signaling was involved in the epithelial plasticity. In Dox-treated, unstimulated cells, *RELA* mRNA was depleted by 90% compared to control consistent with our earlier characterization [28] (Fig. 4 p < 0.01) and the CDE induced *RELA* mRNA expression was reduced by 85% compared to control (Fig. 4, p < 0.01). Importantly, in the RELA KD cells, the CDE-induced expression of SNAI1 and ZEB1 is lost (Fig. 4). Interestingly, the CDH1 induced repression was still observed, indicating that the effect of CDE induced repression was RELA independent (Fig. 4). Collectively these data indicate that RELA controls expression of the core mesenchymal transcription factors in CDE-induced EMT.

### Epithelial RELA directly activates *TGFβ1*

**Fig. 5:**
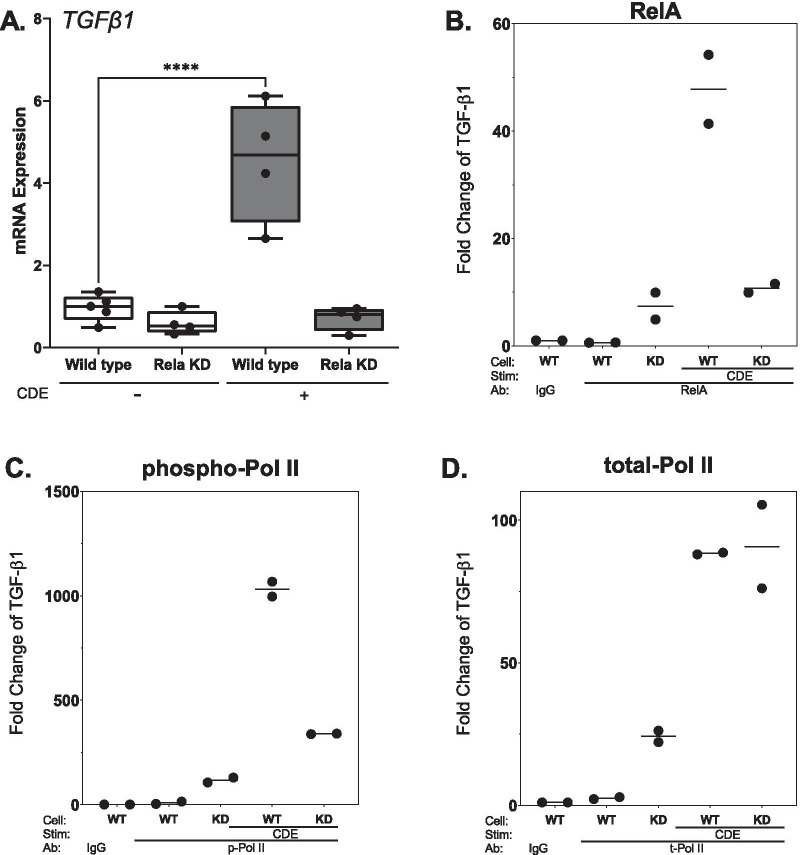
NFκB/RelA signaling directly activates the TGFB1 promoter in response to CDE. **A** DOX regulated RelA shRNA-expressing hSAECs were cultured in the presence of DOX and stimulated treated in the absence or presence of CDE. Abundance of *TGFβ1* mRNA was determined by Q-RT-PCR. **, p < 0.01, post hoc t-test for n = 4–6 biological replicates. **B** Chromatin immunoprecipitation (XChIP) assay was conducted to quantify RELA binding to the proximal *TGFβ1* promoter. Crosslinked chromatin was precipitated using anti-rabbit IgG or anti-RelA and fold enrichment of TGFb1 promoter determined by Q-gPCR. **C** Abundance of phospho-Ser 2 CTD RNA-Pol II on TGFb1 by XChIP. **D** Abundance of Total RNA Pol II; *p < 0.05 compared to Control, t-test. Shown is an individual experiment, reproduced in two independent studies

To directly determine the relationship between NFκB signaling and inducible TGFβ, the RELA WT and KD cells were stimulated in the absence or presence of CDE. We observed that CDE similarly produced a 4.8-fold increase in *TGFβ1* mRNA (p < 0.01, n = 4–6 animals/group). Importantly, the CDE-induced TGFβ1 was strikingly reduced in the RELA KD cells compared to that of control, to levels less than that of unstimulated RELA WT cells (Fig. 5A).

### RELA binds and recruits phosphorylated RNA Pol II to the *TGFβ1* promoter

We next tested the hypothesis that RELA directly activated the *TGFβ1* gene by binding to its regulatory promoter. Cross-linking-Chromatin immunoprecipitation (XChIP) was used to observe the effect of CDE on RELA, and RNA polymerase II [total and phospho-Ser 2 carboxy terminal domain (CTD) RNA -Pol II] binding to the proximal promoter of *TGFβ1*. In WT hSAECs, CDE induced a 50-fold increase in RELA binding to *TGFβ1* (Fig. 5B). Concomitantly, CDE induced a 1000-fold increase in the transcriptional elongation-competent form of phospho-Ser 2 CTD RNA Pol II, and an 80-fold increase in total Pol II (Fig. 5C). By contrast, CDE was unable to increase phospho-Ser 2 CTD RNA Pol II accumulation in the absence of RELA, although the increase in total RNA Pol II was unaffected (Fig. 5D). These data provide direct evidence that CDE induces RELA binding to *TGFβ1* in airway epithelial cells, and downstream recruitment of activated RNA Pol II is dependent on activated RELA.

### RelA signaling in Secretoglobin-expressing (*Scgb1a1*^+^) small bronchiolar cells mediates CDE-induced TGFβ1 expression

To test the role of epithelial RELA in CDE-induced inflammation and remodeling*,* we used an inducible Cre recombinase driven by the bronchiolar epithelial-specific *Scgb1a1* promoter to deplete RelA. In this well-characterized model, *Scgb1a1* is expressed by Club cell progenitor-derived small airway bronchiolar cells [27, 28], a cell type responsible for viral-inducible remodeling and inflammation [42] and sensitization to *Aspergillosis *[17].

**Fig. 6:**
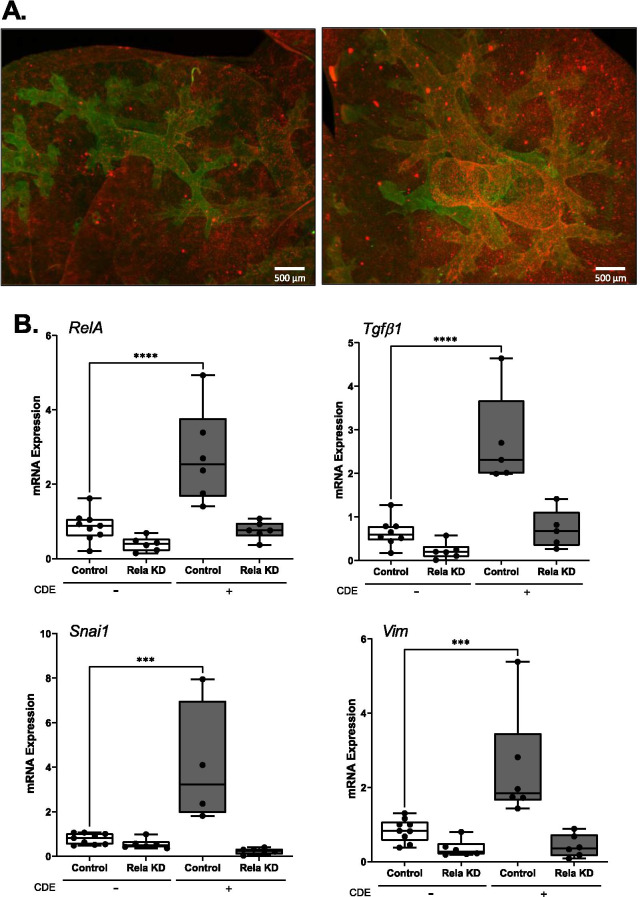
RELA signaling in *Scgb1a1*-expressing small airway cells regulates TGFβ. mTmG x Scgb1a1^CreER^™^/+^  × RelA^fl/fl^ and Scgb1a1^CreER^™^/+^  × RelA^fl/fl^ mice were pre-treated with TMX for 10 days; experiments were conducted 3 wks later. Scgb1a1^CreER^™^/+^  × RelA^fl/fl^ mice were given CDE i.n. over 4 days, n = 5–8. **A** Representative dual-channel maximum intensity projection through 3 mm of tissue depth in a central region of the right lobe. Red-anti-GFP antibody, green-autofluorescence. Scale bar—500 microns. **B** Fold changes in *RelA*, *Tgfβ1, Snai1* and *Vim* mRNAs by Q-RT-PCR of total lung RNA (*p < 0.05 compared to Control, t-test)

Although the expression of SCGB1A1 has been demonstrated by immunofluorescence staining of tissue sections [28] and by FACS analysis [27], the 3 Dimensional distribution of *Scgb1a1* population of cells has not been determined in situ*.* To better understand the distribution of this population of cells, we crossed the *Scgb1a1*^CreER^™^/+^ × RelA^fl/fl^ with a tandem dimer Tomato (mT)-membrane-targeted green fluorescent protein (mG) mouse. All cells in the mT/mG mouse express Tomato, a red fluorescence protein, prior to Cre activation. In response to Cre activation, expression of Tomato is silenced and GFP, a green fluorescence protein, is induced, enabling identification of cells where the *Scgb1a1* promoter is active. We observed TMX-inducible GFP staining in a punctate pattern scattered through small airways and terminal bronchioles into alveoli (Fig. 6A). These data provide the first 3D distribution of Club derived epithelium in the mouse and indicate that this is a distinct spatially located population of innate sensors in the distal airways.

Analysis of *RelA* expression confirmed that TMX administration depletes total lung *RelA* by 50% in the absence of CDE stimulation. Importantly, the threefold increase in *RelA* produced after CDE stimulation was reduced to less than that of untreated WT mice (Fig. 6B). In a manner consistent with the effect of IKKi in wild type mice and RelA siRNA depletion in hSAECs, both basal- and CDE-induced *Tgfβ1* mRNA were substantially reduced to control levels in the RELA KD mice (Fig. 6B). Mesenchymal markers for *Snai1* and *Vim* were also induced in WT mice but substantially reduced in RelA KD mice (0.3 ± 0.34 and 0.6 ± 0.46, respectively p < 0.01, post-hoc t-test, n = 5–8 animals), (Fig. 6B). These findings indicate that the innate pathway controlled mucosal growth factor response and induction of epithelial plasticity (Fig. 6B).

**Fig. 7:**
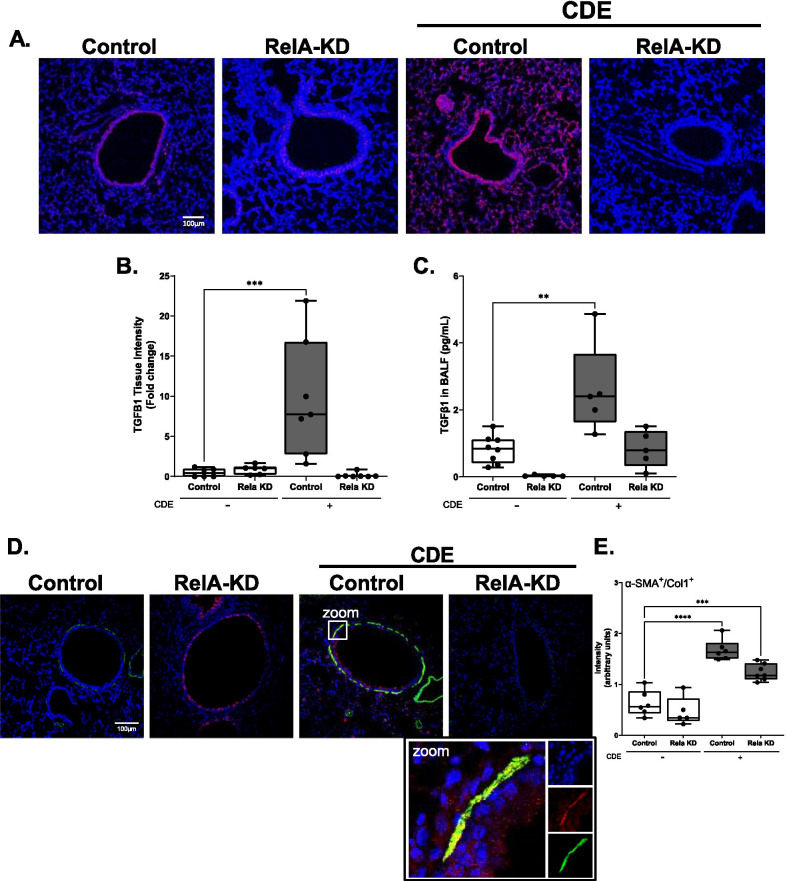
RELA-KD in *Scgb1a1*-expressing epithelial cells reduces CDE-induced TGFβ and myofibroblast expansion. *Scgb1a1*^CreER^™^/+^  × RelA^fl/fl^ mice were pre-treated with TX for 10 days as in Fig. 6 and challenged with/without CDE over 4 d, n = 5–8. **A** IFC. Sections were stained with anti-TGFβ1 Ab (red) and counterstained with DAPI (blue). Images obtained at × 20. **B** Quantitation of TGFβ1 staining. Relative fold changes in FI for each treatment group; *p < 0.01 compared to Control, t-test. **C** Bioactive TGFβ1 in BALF by ELISA. **D** IFC of dual stain between α-SMA (green) and COL1 (red) with blue DAPI blue nuclear staining. Representative images shown at × 20 magnification, with selected enhanced image of the epithelium dissecting the individual colors. **E** Quantification of αSMA + /COL1 + myofibroblasts

The inhibition of *Tgfβ1* mRNA expression was confirmed by reduction of bioactive TGFβ1 protein in the tissue, where a strong induction of TGFβ staining was observed in the small airways and alveoli (Fig. 7A), resulting in a sevenfold increase in fluorescence intensity (Fig. 7B). This staining was lost in both the epithelium and distal alveoli with RELA KD. To confirm that biologically active TGFB1 was being released and regulated by the Club cell NFκB pathway, we analyzed BALF for active TGFB1 by ELISA. A threefold increase in BALF TGFβ1 was observed in the CDE treated mice. Both basal and CDE induced TGFβ1 was inhibited by RELA KD (Fig. 7C). We conclude from these findings that RELA signaling in Club-cell derived cells mediates CDE-induced TGFβ1 response and mesenchymal transition.

### *Scgb1a1-expressing small bronchiolar cells mediates CDE-induced* sub epithelial fibroblast-myofibroblast expansion

Previous challenge experiments in allergic asthmatics have shown that the subepithelial fibroblast population transdifferentiates into myofibrolasts during the late phase of allergic response [43]. As major collagen producers in the airway, myofibroblasts are major contributors to ECM remodeling [44]. However, whether myofibroblasts are induced in naïve airway has not yet been demonstrated. To determine if CDE activates subepithelial myofibroblast populations in naïve animals, myofibroblasts were quantified in the same experiment using an αSMA and Collagen-1 (COL1) dual fluorescent stain. Strikingly, CDE induced a robust activation of αSMA^+^/COL1^+^ myofibroblast population in the subepithelium (Fig. 7D, E). The appearance of this population was blocked by RELA depletion both in control and CDE stimulated conditions. This finding is consistent with the studies that αSMA expression is under control of a TGFβ-regulated element in its proximal promoter [45], and further indicates that RELA activity regulates TGFβ expression and activity, even in the basal state.

## Discussion

Asthma is a global health concern, affecting 235 million individuals worldwide [1]. Allergic asthma is characterized by expansion of the *lamina reticularis* that occurs prior to Th2 polarization and eosinophilia [3]. The detailed mechanisms how aeroallergens induce tissue remodeling are largely unknown. Of relevance to this study, the presence of epithelial plasticity in AA has been well documented, including upregulation of mesenchymal genes in bronchiolar biopsies [6]. In this study, we examine the effect of respiratory tract exposure of a ubiquitous aeroallergen, cat dander, associated with sensitization in > 25% of allergic asthmatics [18]. Here we find that CDE exposure triggers an innate neutrophilic inflammation that transitions to an adaptive macrophage and lymphocytic-rich inflammation over 4 days of exposure. This inflammation is associated with a coordinated inducible mucosal TGFβ expression associated epithelial cell plasticity known as epithelial mesenchymal transition. Our findings that CDE activates the canonical NFκB pathway that drives the epithelial TGFβ1 response is demonstrated through the (a) use of small molecule IKKi inhibitor in wild type mice; (b) NFκB/RelA silencing in *Scgb1a1*-expressing human airway epithelial cells; and (c) NFκB/RelA depletion in *Scgb1a1-*expressing small airway epithelial cells in the mouse. Additionally, we demonstrate that the majority of TGFβ1 protein in the BALF is derived from the *Scgb1a1-*expressing small airway epithelial population and is responsible for expansion of a subepithelial population of αSMA^+^/COL1^+^-expressing myofibroblasts. We therefore conclude that innate NFκB signaling in the Club-derived subpopulation of airway epithelial cells mediates mesenchymal transition and myofibroblast expansion in naïve mice. These findings have important implications for remodeling in allergic asthma.

Aero-allergens are plant and animal products that share the ability to activate mucosal innate signaling cascades through binding TLR receptors as ligands, cleaving PAR receptors or by endogenous NADPH oxidase activity [20, 46, 47]. Our previous work has shown that CDE and the unrelated ragweed pollen share the ability to activate epithelial cells via the TLR4 pathway, in a mechanism distinct from that used by LPS, producing oxidative DNA damage and CXCR2 chemokine expression resulting in neutrophilic inflammation [16, 20, 47]. This study is the first to demonstrate that CDE triggers a coordinated time-dependent increase of all the TGFβ isoforms (TGFβ-1, -2 and -3). These TGFβ isoforms are members of a superfamily of injury-response and growth factors controlling cell cycle regulation, cellular differentiation and collagen deposition/fibrogenesis [48]. It is well-established exacerbations trigger secretion of epithelial TGFβ and other growth factors in sensitized individuals [49]. However, it is difficult to separate out contributions from Th2/eosinophils from those of the epithelium. Our studies in naïve mice suggest that CDE can trigger mucosal TGFβ injury-repair processes directly.

Although TGFβ release is also regulated at the post-translational level [50], our findings that increased TGFβ1 protein in the BALF is inhibited by disrupting NFκB signaling strongly suggests to us that *inducible* mucosal *Tgfβ1* expression mediates airway remodeling, consistent with the findings of others in the HDM model [51], and viral models [27, 52]. In addition, the finding that SMAD3 activation is in the mucosa indicates that airway epithelial cells themselves are a major target of this secreted TGFβ. In normal epithelial cells, TGFβ induces type II EMT, a cellular state associated with loss of epithelial polarity, enhanced mobility and ECM remodeling [24, 37], driven by expression of SNAI1 and ZEB1 [53]. Although EMT is recognized to be a highly dynamic state characteristic of epithelial cell plasticity [54], the SNAI1 and ZEB1 autoregulatory circuit is a hallmark of the stable transition. Our studies are the first, to our knowledge, demonstrating that CDE results in the activation of the SNAI1/ZEB1 core transcription factors.

Epithelial plasticity is a multi-step process from sequential expression of gene expression networks under master transcription factor control [55]. Recent work by our group has shown that the RELA transcription factor is upstream of viral- and TGFβ-induced cell state changes [30]. Our study extends these earlier findings showing that CDE-induced RELA binding to *TGFβ1* is required for accumulation of Ser2-phosphorylated RNA Polymerase II. Ser2 phosphorylation is a key regulatory switch in gene expression, enabling inactive RNA Pol II to acquire processive properties to transcribe full-length *TGFβ1* mRNA [56, 57]. Previously we were able to show that RELA promoter binding induces Ser2-phosphorylated RNA Pol II by recruiting the chromatin-modifying CDK9∙BRD4 complex [56, 58, 59]. More work will be required to understand the role of the CDK9∙BRD4 complex in the innate mucosal response to aeroallergens.

Another major finding is the discovery of the direct signaling role of *Scgb1a1-*expressing epithelial cells in the TGFβ1 response to CDE. *Scgb1a1*^+^ Club cells are one of five major small airway epithelial cell types that have been identified by single cell sequencing and cell lineage studies [60, 61]. Lineage inference suggests that club cells differentiate into goblet and multiciliated cells, enriched in the distal airway [60, 61] playing major roles in injury/repair in the small airways/terminal bronchioles [62]. Despite the understanding of the cellular lineage, the native 3D distribution of these cells has been elusive. Our lung clearing immunofluorescence light sheet microscopic imaging provides the first evidence that this population of innate mucosal “sensors” are distributed throughout the small airways and terminal bronchioles. Previous work has shown that inducible TGFβ1 expression by *Scgb1a1*^+^ cell population is important for luminal recruitment of TGFBRII -expressing IL13 + innate lymphoid cells and airway hyperreactivity [51]. Our study extends this work to demonstrate that RelA signaling is directly *upstream* of TGFβ1 expression, necessary for inducible TGFβ expression, linking NFκB to epithelial TGFβ1 response to alveolar TGFB1 expression and subepithelial myofibroblast transdifferentation. We interpret our findings that the alveolar TGFβ expression is dependent on Club cell TGFβ as a manifestation of the well-known TGFβ autocrine loop, where the presence of TGFβ stimulates its own expression. The myofibroblast population is dynamic, increasing during the late phase of allergic inflammation [43], refractory asthma [63], and recurrent asthma [64]. Our data indicates that this population expands in response to naïve CDE exposures mediated by epithelial cell RELA signaling.

## Conclusions

In summary, we report the discovery that key innate Club-cell derived epithelial “sensors” in the small airways trigger TGFβ activation through RELA, resulting in epithelial plasticity and myofibroblast expansion. These cellular responses account for the disruption of mucosal barrier and remodeling of the *lamina retiularis* foundational to mucosal remodeling in allergic lung disease.

## Supplementary Information

**Additional file 1: Table S1.** PCR Primers. **Figure S1.** Percentage of live cells from BAL fluid of mice treated for 4 days with CDE, n=6-8. **Figure S2.** C57BL/6J mice were treated with daily challenges of CDE or PBS i.n. over 4 d. Immunofluorescence microscopy (IFM). Sections were stained with (a) anti-TGFβ1, (b) anti-RelA, and (c) anti-Snail antibody (Ab, red color) and counter-stained with DAPI (blue) to visualize nuclei; n = 6-8 animals/group. **Figure S3.** Single cell flow cytometry using a 4-day CDE exposure model. Representative gating for cell selection using Fluorescence Minus One (FMO) controls to select the correct gate for SMA+ and CD326+ CDE-exposed cells; n=5. **Figure S4.** C57BL/6J mice were treated with daily challenges of CDE or PBS i.n. and given BMS-345541 (IKKi) i.p. simultaneously over 5 d, n=5-6. A. IFM. Sections were stained with anti p-SMAD3 Ab (red) and DAPI (blue). **Figure S5.** Scgb1a1CreERTM/+ × RelAfl/fl mice were pre-treated with TX for 10 d and challenged with/without CDE over 4 d, n=5-8. A. IFC of dual stain between α-SMA (green) and Collagen I (purplered) with blue DAPI blue nuclear staining B. IFC Sections were stained with anti-TGFβ1 Ab (red) and counterstained with DAPI (blue). Images obtained at 20X.

## Data Availability

Not applicable.

## Abbreviations

AA: Allergic asthma
BALF: Bronchoalveolar lavage fluid
CDE: Cat dander extract
CDH1: Cadherin 1
COL1: Collagen 1
ECM: Extracellular matrix
FI: Fluorescence Intensity
IKK: IκB kinase
NFκB: Nuclear factor-κB
RELA: V-rel avian reticuloendotheliosis viral oncogene homolog A
αSMA: Smooth muscle cell actin, α chain
Scgb1a1: Secretoglobin family 1A Member 1
SMAD3: SMAD Family Member 3
SNAI1: Snail Family Transcriptional Repressor 1
TGFβ: Transforming growth factor β
TLR: Toll-like receptors
TMX: Tamoxifen
XCHIP: Two step chromatin immunoprecipitation
ZEB1: Zinc Finger E-Box Binding Homeobox 1
