# Supplementary material for: Role of Secretoglobin+ (club cell) NFκB/RelA-TGFβ signaling in aero-allergen-induced epithelial plasticity and subepithelial myofibroblast transdifferentiation
Source: Respir Res. 2021 Dec 20;22:315. doi: 10.1186/s12931-021-01910-w (PMC8690490; doi:10.1186/s12931-021-01910-w)
Supplement: Supplementary file 1 — Additional file 1: Table S1. PCR Primers. Figure S1. Percentage of live cells from BAL fluid of mice treated for 4 days with CDE, n=6-8. Figure S2. C57BL/6J mice were treated with daily challenges of CDE or PBS i.n. over 4 d. Immunofluorescence microscopy (IFM). Sections were stained with (a) anti-TGFβ1, (b) anti-RelA, and (c) anti-Snail antibody (Ab, red color) and counter-stained with DAPI (blue) to visualize nuclei; n = 6-8 animals/group. Figure S3. Single cell flow cytometry using a 4-day CDE exposure model. Representative gating for cell selection using Fluorescence Minus One (FMO) controls to select the correct gate for SMA+ and CD326+ CDE-exposed cells; n=5. Figure S4. C57BL/6J mice were treated with daily challenges of CDE or PBS i.n. and given BMS-345541 (IKKi) i.p. simultaneously over 5 d, n=5-6. A. IFM. Sections were stained with anti p-SMAD3 Ab (red) and DAPI (blue). Figure S5. Scgb1a1CreERTM/+ × RelAfl/fl mice were pre-treated with TX for 10 d and challenged with/without CDE over 4 d, n=5-8. A. IFC of dual stain between α-SMA (green) and Collagen I (purplered) with blue DAPI blue nuclear staining B. IFC Sections were stained with anti-TGFβ1 Ab (red) and counterstained with DAPI (blue). Images obtained at 20X. [file 12931_2021_1910_MOESM1_ESM.pdf]

Supplemental Methods

Name	Primer Sequence (mouse)
**gadph**	Forward: CAGATCCACGACGGACACATTGGG
	Reverse: CATGACAACTTTGGCATTGTGG
**tgfb1**	Forward: CAACAATTCCTGGCGTTACCTTGG
	Reverse: GAAAGCCCTGTATTCCGTCTCCTT
**tgfb2**	Forward: CTGTCTACCTGCAGCACACT
	Reverse: TGGGACTGTCTGGAGCACAA
**tgfb3**	Forward: AGCATCCACTGTCCATGTCA
	Reverse: TTCTTCCTCTGACTGCCCTG
**smad3**	Forward: CCAGCACACAATAACTTGGA
	Reverse: AGACACACTGGAACAGCGGA
**rela**	Forward: AAG AGC AGC GTG GGG ACT AC
	Reverse: TGC CAG AGT TTC GGT TCA CT
**snail1**	Mm00441533_g1
**zeb1**	Mm00495564_m1
**vim**	Mm01333430_m1
**ppia**	Mm02342430_g1
**Name**	**Primer Sequence (human)**
**PPIA**	Hs04194521_s1
**TGFb1**	Hs00998133_m1
**RelA**	Hs01042014_m1
**SNAIL1**	Hs00195591_m1
**ZEB1**	Hs01566408_m1
**CDH1**	Hs01023895_m1

Supplementary Table S1: PCR Primers.

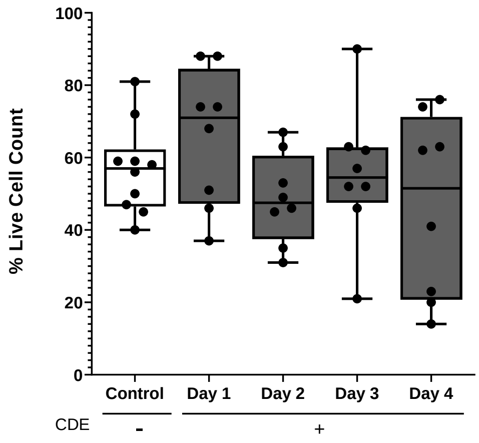

Supplementary Figure 1: Percentage of live cells from BAL fluid of mice treated for 4 days with CDE, n=6-8.

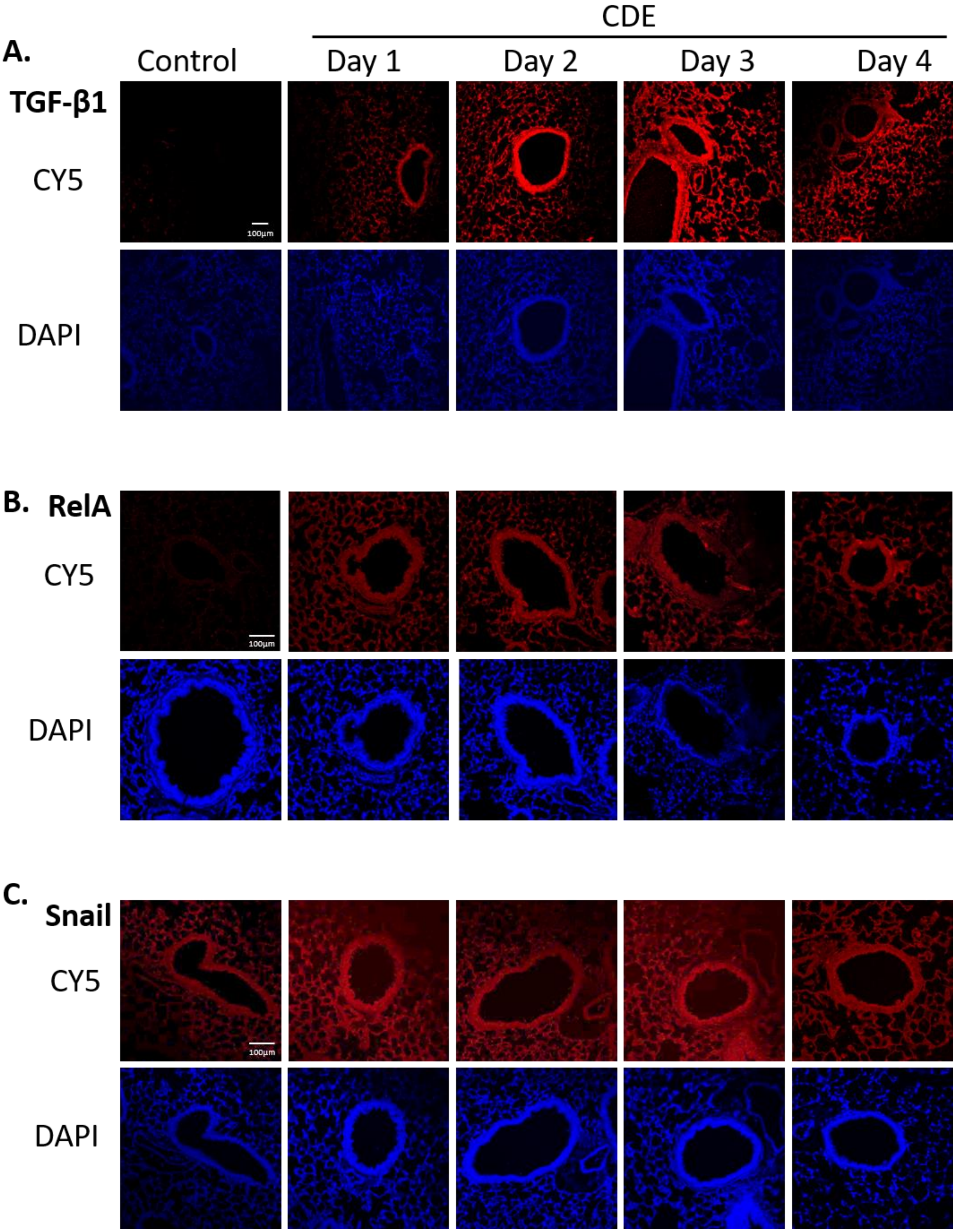

Supplementary Figure 2: C57BL/6J mice were treated with daily challenges of CDE or PBS i.n. over 4 d. Immunofluorescence microscopy (IFM). Sections were stained with (a) anti-TGFβ1, (b) anti-RelA, and (c) anti-Snail antibody (Ab, red color) and counter-stained with DAPI (blue) to visualize nuclei; n = 6-8 animals/group

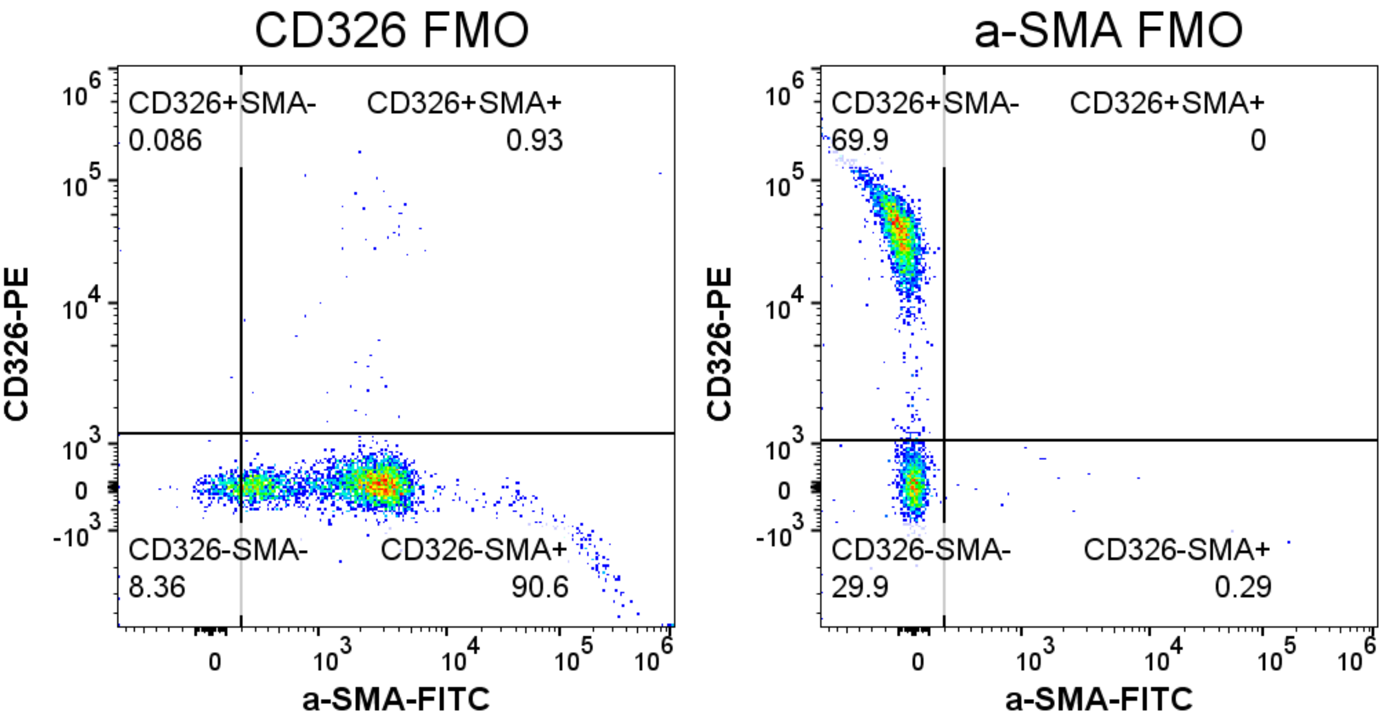

Supplementary Figure 3: Single cell flow cytometry using a 4-day CDE exposure model. Representative gating for cell selection using Fluorescence Minus One (FMO) controls to select the correct gate for SMA$^+$ and CD326$^+$ CDE-exposed cells; n=5.

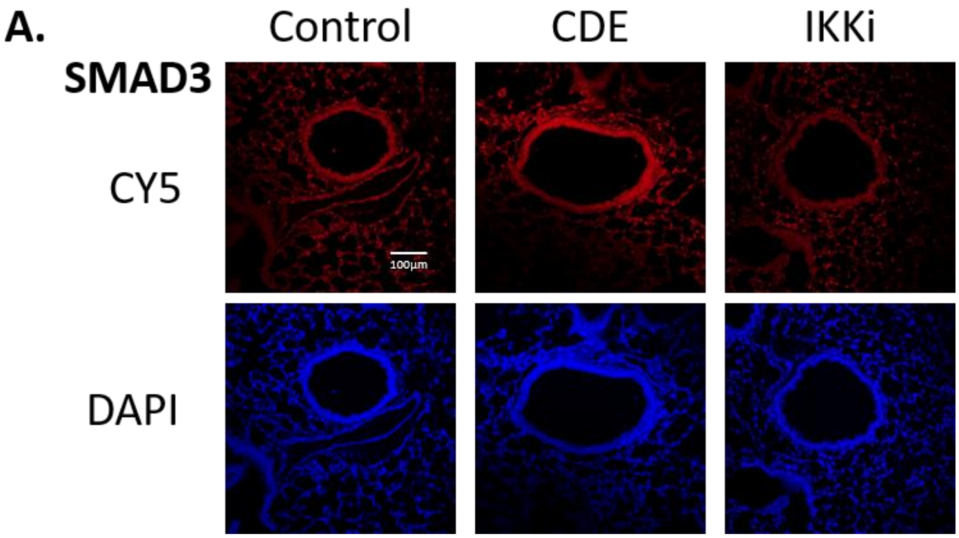

Supplementary Figure 4: C57BL/6J mice were treated with daily challenges of CDE or PBS i.n. and given BMS-345541 (IKKi) i.p. simultaneously over 5 d, n=5-6. A. IFM. Sections were stained with anti p-SMAD3 Ab (red) and DAPI (blue).

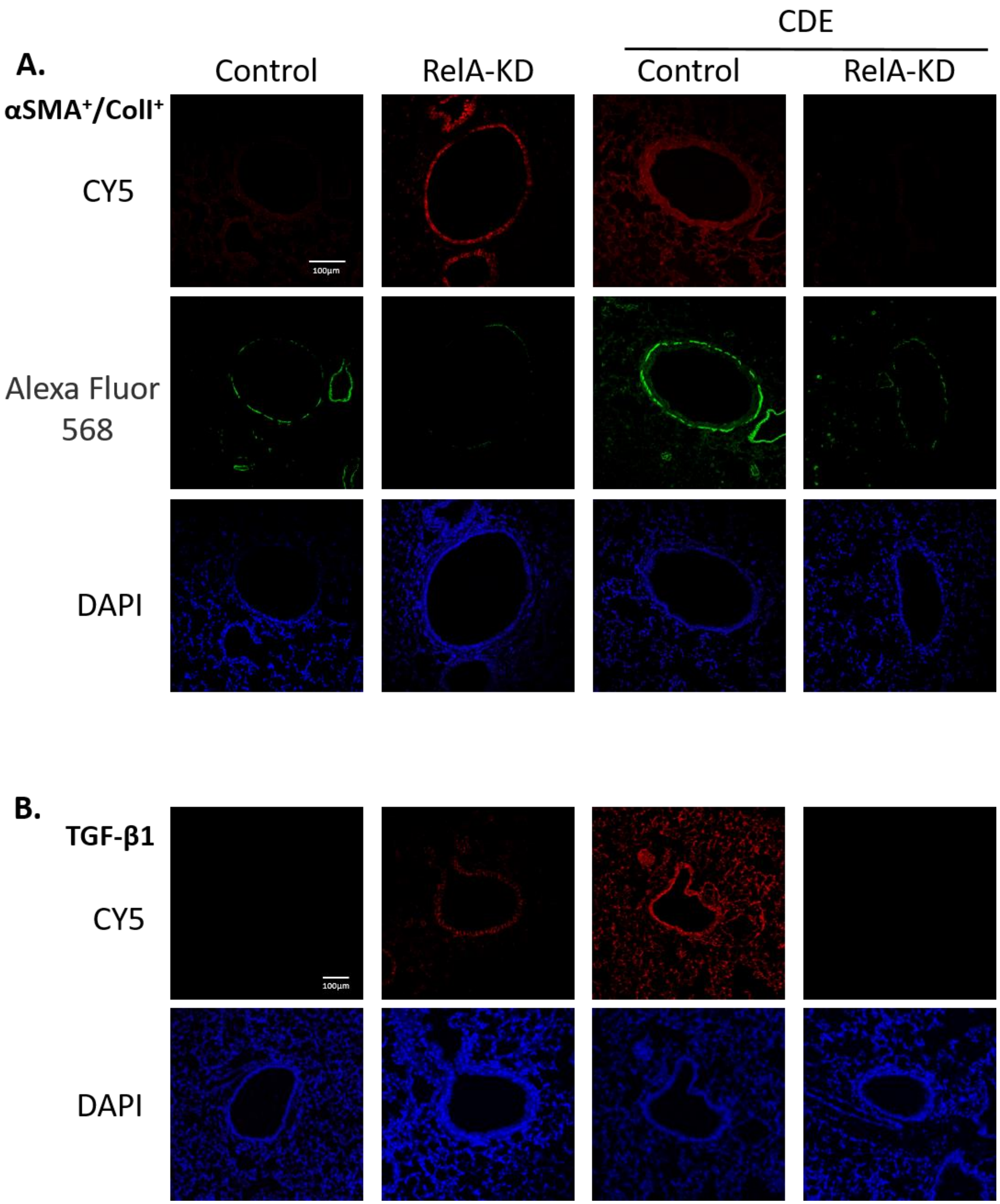

Supplementary Figure 5: Scgb1a1CreERTM/+ × RelAfl/fl mice were pre-treated with TX for 10 d and challenged with/without CDE over 4 d, n=5-8. A. IFC of dual stain between α-SMA (green) and Collagen I (purplered) with blue DAPI blue nuclear staining B. IFC Sections were stained with anti-TGFβ1 Ab (red) and counterstained with DAPI (blue). Images obtained at 20X.
